# The soldiers needed to be awakened: Tumor-infiltrating immune cells

**DOI:** 10.3389/fgene.2022.988703

**Published:** 2022-09-29

**Authors:** Wang Yaping, Wang Zhe, Chu Zhuling, Li Ruolei, Fan Pengyu, Guo Lili, Ji Cheng, Zhang Bo, Liu Liuyin, Hou Guangdong, Wang Yaoling, Hou Niuniu, Ling Rui

**Affiliations:** ^1^ Department of Thyroid, Breast and Vascular Surgery, Xijing Hospital, Fourth Military Medical University, Xi’an, China; ^2^ Department of General Surgery, Eastern Theater Air Force Hospital of PLA, Nanjing, China; ^3^ Department of Urology, Xijing Hospital, Fourth Military Medical University, Xi’an, China; ^4^ Department of Geriatrics, Union Hospital of Tongji Medical College, Huazhong University of Science and Technology, Wuhan, China

**Keywords:** tertiary lymphoid structures, antigen presentations, immunotherapy, tumor-infiltrating immune cells, tumor microenvironment

## Abstract

In the tumor microenvironment, tumor-infiltrating immune cells (TIICs) are a key component. Different types of TIICs play distinct roles. CD8+ T cells and natural killer (NK) cells could secrete soluble factors to hinder tumor cell growth, whereas regulatory T cells (Tregs) and myeloid-derived suppressor cells (MDSCs) release inhibitory factors to promote tumor growth and progression. In the meantime, a growing body of evidence illustrates that the balance between pro- and anti-tumor responses of TIICs is associated with the prognosis in the tumor microenvironment. Therefore, in order to boost anti-tumor response and improve the clinical outcome of tumor patients, a variety of anti-tumor strategies for targeting TIICs based on their respective functions have been developed and obtained good treatment benefits, including mainly immune checkpoint blockade (ICB), adoptive cell therapies (ACT), chimeric antigen receptor (CAR) T cells, and various monoclonal antibodies. In recent years, the tumor-specific features of immune cells are further investigated by various methods, such as using single-cell RNA sequencing (scRNA-seq), and the results indicate that these cells have diverse phenotypes in different types of tumors and emerge inconsistent therapeutic responses. Hence, we concluded the recent advances in tumor-infiltrating immune cells, including functions, prognostic values, and various immunotherapy strategies for each immune cell in different tumors.

## Introduction

Immunotherapies have become increasingly important for tumor patients, particularly those with advanced tumors (Tarantino et al., 2022). It is well known that using immune checkpoint blockades (ICBs) has yielded a beneficial effect in patients with advanced melanoma and lung cancer (Mehdizadeh et al., 2021); adoptive cell therapies (ACT) and chimeric antigen receptor (CAR)-T cells therapy have also improved the prognosis of patients with hematologic tumors (Martinez and Moon, 2019). However, immunotherapy resistance occurs in some tumors, and a possible explanation for this condition is the complication of the tumor microenvironment (TME) (Whiteside, 2012). TME, which is created by various cells and soluble molecules including immune cells and cytokines, exerts significant effects on tumor development and progression (Duhan and Smyth, 2021). In TME, the crosstalk of immune cells and tumor cells significantly controls tumor growth, namely, cancer immunoediting (Burnet, 1970). Cancer immunoediting involves three phases: elimination, equilibrium, and escape (Dunn et al., 2002; Wilczyński and Nowak, 2012). In the elimination phase, various effector cells and molecules destroy tumor cells and dampen tumor progression. For instance, dendritic cells (DCs) can present tumor antigens to T cells, and subsequently, T cells release perforin and granzyme to inhibit tumor cell growth or kill tumor cells through the Fas/FasL signal pathway. However, if immune cells can not eliminate tumor cells, cancer immunoediting might proceed into the equilibrium or escape phase. In the equilibrium phase, tumor cells could not be detectable and are deemed to be in a dormant status in the clinical. However, when the balance between tumor proliferation and apoptosis is disturbed by various signaling pathways, like the Wnt/β-catenin pathway, tumor cells start to proliferate dramatically and result in tumor metastasis, namely, the escape phase (Wilczyński and Nowak, 2012). In the escape phase, the anti-tumor immune response is weakened or suppressed *via* multiple mechanisms which mainly disturb the cancer immunity cycle (Wilczyński and Nowak, 2012; Wada et al., 2022). The cancer immunity cycle also consists of three phases: priming, migration, and effector. In the priming phase, the process of antigen-presenting is hampered by inhibitory signaling pathways, which impairs the activation of effector cells. In the migration phase, tumor cells release inhibitory molecules to restrain immune cell infiltration. In the effector phase, these mechanisms are even more complex. Immune cells infiltrating into the tumor sites perform diverse functions, thus, they influence tumor progression in various ways. The function of these immune cells will be discussed below (Wada et al., 2022). Importantly, immune checkpoints (ICs) are essential for tumor progression in every phase. Over the past decades, attention given to ICs has increasingly grown. The ICs can be produced by various cells, including immune cells and tumor cells infiltrating the TME. They could cause the dysfunction of effector cells and inhibit the apoptosis of tumor cells (Mehdizadeh et al., 2021; Munari et al., 2021). Apart from the immune cell components, cancer-associated fibroblasts (CAFs) and tumor endothelial cells (ECs) are associated with an aberrant vascular system that can transport nutrition to tumor cells and disturb the therapeutic delivery of T cells into the tumor sites (Nagarsheth et al., 2017; Lamplugh and Fan, 2021). It is well known that high demands for nutrients in tumor cells lead to the formation of abnormal vascular networks which promote tumor growth. Due to the intense competition for nutrients between tumor cells and immune cells, the nutrients and oxygen are insufficient in TME, causing a hypoxic and acidic status. Hypoxia-inducible factor 1-alpha (HIF1α) is a key factor in upregulating the level of vascular endothelial growth factor (VEGF) that arms the aberrant vasculature and fosters the epithelial-–mesenchymal transition (EMT) in the hypoxic microenvironment (Lamplugh and Fan, 2021). Under the hypoxic condition, tumor cells could escape immunosurveillance depending on activated HIF1α signaling which promotes CTL apoptosis. Besides, in TME, tumor cells and other immunosuppressive cells could express indoleamine 2,3-dioxygenase (IDO), which depletes tryptophan and results in the impairment of CD8+T cell cytotoxicity (Lamplugh and Fan, 2021). Other substances metabolized by tumor cells, including hyper glycolysis, lactate, and lipid, can impede the antigen-presenting process of DCs, recruit regulatory T cells (Tregs), and help tumor cells eventually escape from immune surveillance (Davis et al., 2015). Additionally, soluble factors also deliver signals to control tumor development. For example, upon the high levels of tumor-derived lactate, high-expressed PD-L1 on the surface of tumor cells, or IL-4, IL-10, and TGF-β are present in TME, tumor-associated macrophages (TAMs) would polarize into the M2 phenotype, which plays a pro-tumor role (Goossens et al., 2019; Petty et al., 2019). The presence of TGF-β in TME also stimulates TAMs to produce arginase-1 (Arg-1) and inhibit T cell immune response. Hereby, since the complex TME controls the benefits of immunotherapy, a comprehensive understanding of the complex components of tumor-infiltrating immune cells is required for tumor immunotherapy. In this review, we discussed the role of tumor-infiltrating immune cells in the process of tumor elimination in TME, as well as current immunotherapeutic strategies. In addition, we described the function and predictive value of tertiary lymphoid structures in TME.

## Priming phase

Tumor antigens could be recognized by DCs, which present antigens to T cells and activate T cells. This process is a pivotal step in the priming phase (Eryn and Ott, 2021). Tumor antigens include tumor-specific antigens (TSAs) and tumor-associated antigens (TAAs). Tumor antigens include mutant and viral antigens. Genomic aberrations of tumor cells result in mutant antigens, which affects antigens recognition and presentation (Lu et al., 2014). Therefore, a comprehensive understanding of the antigen-presenting cells (APCs) is extremely critical.

### Dendritic cells

DC subsets are specialized in antigen recognition and presentation and induce a tumor-specific immune response in patients. DC subsets are divided into conventional dendritic cells (cDCs), plasmacytoid DCs (pDCs), and monocyte-derived DCs (moDCs), according to different functions and phenotypes (Kvedaraite and Ginhoux, 2022). Notably, cDCs include are of two types: type 1 (cDC1s) and type 2 (cDC2s). cDC1s are critical for anti-tumor response and are associated with patient survival. cDC1 infiltration apparently improved prognosis in solid tumors, such as head and neck squamous cell carcinomas (HNSCC) , lung adenocarcinoma, melanoma, and triple-negative breast cancer (TNBC) (Bogunovic et al., 2009; Roberts et al., 2016; Barry et al., 2018; Böttcher et al., 2018). cDC1s express XC-chemokine receptor 1 (XCR1), which is used to make a distinction between cDC1 and other DC subsets (Villani et al., 2017). XCR1 expressed by cDC1s could bind to the CD8+T cell phenotype XC-chemokine ligand 1 (XCL1), which activates T cell function. XCL1 is also expressed by tumor cells, which boosts this process by activating T cells (Matsuo et al., 2018; Sánchez-Paulete et al., 2018; Ferris et al., 2020). CD103+ cDCs1 can prime CD8+ T cells and CD4+T cells by cross-presenting antigen (Cancel et al., 2019). CD40 expressed by cDCs1 binds to the CD40 ligand, which is produced by CD4+T cells, which activates CD8+T cells (Schoenberger et al., 1998). cDC1s also express CXC-chemokine ligand 9(CXCL9) and CXCL10 to activate CXCR3+ T and NK cells, recruit CD8+ T cells into the tumor sites, and foster the efficacy of anti-PD-1 or anti-TIM-3 therapy (de Mingo Pulido et al., 2018; Chow et al., 2019). Moreover, after the use of PD-1 blockade, CD8+T cells release IFN-γ, which promotes cDC1 to secrete IL-12 by using the non-canonical NFκB-dependent mechanism. In turn, IL-12 augments CD8+T cell functions (Stratikos et al., 2014; Garris et al., 2018). As a side note, the primary source of the CXCL9 and CXCL10 seems to be expressed by CD103+cDC1s in TME (Mikucki et al., 2015). Additionally, CCL5 and Fms-related tyrosine kinase 3 (FLT3) produced by NK cells or CCL4 produced by tumor cells could attract cDC1s into the tumor sites (Barry et al., 2018; Böttcher et al., 2018), but the activation of the WNT/ β-catenin signaling pathway and the accumulation of prostanoidprostaglandinE-2(PGE2) in TME could deduce the production of CCL4/CCL5, respectively (Spranger et al., 2017; Böttcher and Reis e Sousa, 2018; Ruiz de Galarreta et al., 2019).

cDC2s are specialized in priming CD4+T cells through MHC-II molecules and secreting IL-12 (Mittag et al., 1950; Segura et al., 2013; Jhunjhunwala et al., 2021). When Tregs are depleted, cDC2s could potently activate CD4+T cells to kill tumor cells and are associated with a favorable prognosis in HNSCC and melanoma (Binnewies et al., 2019). However, the functions of cDC2 in TME are less clear. The function of pDCs is complicated for controlling tumor progression. pDCs may promote tumor growth, foster angiogenesis, and promote metastasis in TME by triggering Tregs and releasing inducible co-stimulator (ICOS)-L, PD-L1, and IDO (Aspord et al., 2013). Some studies have shown that higher pDC frequencies are correlated with worse outcomes (Kvedaraite and Ginhoux, 2022). Conversely, pDCs also play the anti-tumor role by producing type I interferons (IFN-Is), which enhances the cytotoxicity of T cells and NK cells, or releasing Granzyme B that kills tumor cells directly. In TME, some inhibitory factors, like TGF-β, could also impair toll-like receptor (TLR)–induced IFN-α secretion by pDCs and promote tumor growth (Kvedaraite and Ginhoux, 2022). Notably, the pDC functions in cross-priming CD8+T cells remain currently unclear and need to be further dissected (Fu et al., 2022). At this juncture, it is well documented that moDCs are the inflammation subsets and produce various inflammatory cytokines to induce tumor growth (O'Keeffe et al., 2015). On the contrary, moDCs loading tumor antigens inhibit tumor progression by cross-presenting antigens, and this property has been considered as a therapeutic agent (Ma et al., 2013). However, the function of moDCs is still thoroughly unclear in tumor settings (Duhan and Smyth, 2021; O'Keeffe et al., 2015). Lastly, several factors in the tumor microenvironment have been implicated in the evolution of DCs into a tolerogenic phenotype, including TGF-β, IL- 10, and VEGF. This tolerogenic property of DCs might help tumor cells escape from immune surveillance, limit effector T cells functions, boost the production and expansion of Tregs, and even induce DC apoptosis (Mahnke et al., 2003; Chen et al., 2017; Castenmiller et al., 2021).

Furthermore, antigen presentation can also be influenced by tumor cells. During tumor development, tumor antigens can be lost or mutated, leading to the formation of neoantigens. Even with the assistance of HSP90, neoantigens are hidden by the tumor and result in the dysfunction of DCs (Jaeger et al., 2019). A study has shown that tumor antigen loss was associated with resistance to ICB in non-small small-cell lung cancer (NSCLC) (Anagnostou et al., 2017). Expression of the HLA-I complex is reduced by genetic alterations and the modulation of transcription, failing to recognize antigens (Jhunjhunwala et al., 2021). Cytokines also affect the expression of the HLA-​I complex. For instance, the inhibition of IFN-γ signaling pathways decreases the level of the HLA-I complex and leads to resistance to anti-CTLA-4 therapy in melanoma (Gao et al., 2016). However, while the deficiency is tumor-specific, how does an immune response recognize antigens? This issue requires an in-depth research (Jhunjhunwala et al., 2021).

*DC-based immunotherapies*: Given the properties of DCs and tumor antigens, using the cDC1-based vaccine in mice tumor can enhance infiltration of T cells and halt tumor progression (Wculek et al., 2019). It was discovered that targeting XCR1 is crucial for the delivery of tumor antigen to cDC1 and, subsequently, CD8+T cell priming (de Mingo Pulido et al., 2021). The cDC2-based vaccine may also potently inhibit tumor growth and prolong the survival (Saito et al., 2022). Treatment with antibodies against the CD47-SIRPα axis could activate cDC2s, enhancing the cytotoxicity of CD8+ T cells (Saito et al., 2022). FLT3 is a key factor for the differentiation and maturation of cDCs; thus, FLT3 agonist, CDX-301 (FLT3L), has been developed (Kvedaraite and Ginhoux, 2022). A study has reported that FLT3L boosts the efficacy of DC-targeting vaccines in melanoma (Bhardwaj et al., 2020). Besides, some studies for other tumors are under the clinical trials (NCT04491084, NCT05029999, and NCT05010200). In recent years, pDCs-based treatment has been developed and has acquired benefits. For instance, using vaccination based on pDCs could enhance CD8+ T functions and improve the prognosis of patients (Tel et al., 2013; Westdorp et al., 2019) (NCT01863108). Additionally, the TLR7/TLR8 agonists used to activate pDCs are currently in preclinical models (Zhou et al., 2022) and are under clinical trials (NCT04588324, and NCT03906526). MoDCs-based vaccines have been generated, which loads tumor (neo)antigens for presentation to T cells. MoDCs-based vaccines can overcome the “silence” of DCs caused by neoantigens to restore and enhance the presentation functions of DCs, and improve the prognosis in melanoma patients (Carreno et al., 2015). It is well documented that using autologous monocyte-derived DC vaccination could facilitate the cytotoxicity of CD8+ T cells (Baek et al., 2015) (NCT02285413). Researchers have also shown autologous DC-based vaccines in which tumor antigens are loaded could also be considered as a potential therapeutic strategy through delivering the antigen presenting cells (Yewdall et al., 2010). Another study has shown that small interfering RNA (siRNA) reduces cDC1-immunosuppressive signals to delete PD-L1 and PD-L2 from moDCs (Hobo et al., 2010). A DCs-based vaccine combined with CTLA-4 inhibitor enhanced anti-tumor response (Ribas et al., 2009). Treatment with TLR9 agonists and anti-PD-1 was also associated with a high infiltration of DCs (Ribas et al., 2018). Furthermore, nanomaterials with autophagy regulation have been developed, which is important for DC function and facilitates its anti-tumor activity (Guan et al., 2022). Engineered exosomes to activate DCs have also been proposed and are considered as a promising method to develop (Huang et al., 2022a; Fu et al., 2022). For instance, HELA-Exos play an anti-tumor role by activating cDC1 and then enhancing the function of CD8+ T in breast cancer (Huang et al., 2022a). Despite the fact that DC vaccines have acquired good efficacy in mouse models and clinical trials, they still face huge challenges as a treatment strategy, as DC vaccines could not be appropriate for a wide range of cancers.

### B cells

B cells could also take up antigens and process antigens by MHC class molecules to T cells (Avalos and Ploegh, 2014; Bruno et al., 2017). Extensive infiltration of B cells promotes tumor antigens to stimulate T cells potently and is associated with longer progression-free survival (PFS) and overall survival (OS) in NSCLC (Germain et al., 2014). B cells exert an important influence which activates CD4+T cells and induces CD4+T cell differentiation into follicular helper T (Tfh) cells (Hong et al., 2018). CD40L on activated T helper cells binds to CD40 on B cells to promote proliferation and development of B cells, and B cells and Tfh cells are involved in the formation of germinal centers (GCs) (He et al., 2013; Crotty, 2019). Intratumoral B cells could differentiate into plasma cells that express CD38, CD138, and CD79a. In high-grade serious ovarian cancer, the presence of high-level CXCL-13 +B cells, T cells, and PCs signified a better survival (Kroeger et al., 2016; Montfort et al., 2017; Moran et al., 2021). Intratumoral B cells switch isotypes and produce IgG or IgA antibodies, which is contradictory in influencing tumor growth (Lauss et al., 2021). Lastly, regulatory B (Breg) cells have been proposed in TME (Saze et al., 2013). Breg cells could produce TGF-β, IL-10, and IL-35, facilitate Treg polarization and help M2 macrophages and myeloid-derived suppressor cells (MDSCs), leading to disturbing tumor antigen presentation and promoting tumor proliferation. CD39 and CD73 on the surface of Breg cells could hydrolyze ATP to adenosine and suppress the tumor death in TME (Brossart, 2022; Flores-Borja and Blair, 2022). Therefore, the role of B cells is a double-edged sword (Fridman et al., 2020).

### B-based immunotherapies

Some studies have proved that the fusion of antigen peptides loading on B cells can further enhance anti-tumor immune efficacy. The CD40/CD40L pathway is also critical to adoptive cell therapies with tumor antigen peptide-loaded B cells (Evans et al., 1950; Wennhold et al., 2017). Furthermore, ACT with CD40-activated B cells loaded with RNA encoding tumor antigen or DNA encoding tumor antigen inhibited the progression of melanoma and colorectal cancer (Gerloni et al., 2004; Colluru and McNeel, 2016). B-cell receptor (BCR) on the surface of B cells can directly process antigens and activate T cells. Thus, researchers exploited this trait to edit a specific BCR toward tumor antigens *in vitro*. The editing BCR strategies are attractive, but they have are yet to be applied to treat tumors (Page et al., 2021). Antibodies, targeting B cells, are mainly used to treat hematological malignancy, such as anti-CD19 and anti-CD20, which results in a conducive prognosis (NCT04160195), and currently, relevant trials are on the way.

## Migration phase

Activated T cells primarily eliminate tumor cells in TME. Hence, activated T cells need to migrate from blood vessels to the microenvironment with the influence of various molecules and constructions. Vascular endothelial growth factor expressed by tumor cells can promote tumor angiogenesis and inhibit the migration of activated T cells (Nagarsheth et al., 2017). Adhesion molecules, including intercellular adhesion molecule-1 (ICAM-1) and vascular cell adhesion molecule-1 (VCAM-1), could help T cells to adhere to the vessel wall and migrate into TME (Lamplugh and Fan, 2021). Recently, ectopic lymphoid aggregation has been discovered in the tumor sites, which resembles secondary lymphoid organs (SLOs), termed tertiary lymphoid structures (TLSs) (Dieu-Nosjean et al., 2008). High endothelial venules (HEVs), one of the components of TLS, can facilitate the migration of immune cells into the tumor sites and accelerate tumor cell destruction (Sautès-Fridman et al., 2019). Lymphoid tissue-inducer cells (LTi), initiating SLOs formation, may enhance the expression of adhesion molecules like VCAM1 and ICAM1, and then stimulate HEV formation of TLS by expressing LTα1β2, which could combine with LTβ. However, it is still unclear whether LTi cells drive TLS formation (Jacquelot et al., 2021a; Schumacher and Thommen, 2022).

Consequently, promoting immune cell migration into the TME could be a usable strategy to enhance anti-tumor immunity. Several studies have demonstrated that the combination of anti-PD-L1 and antiangiogenic therapy can facilitate intratumoral HEV formation and augment the efficacy of immunotherapies (Allen et al., 2017; Johansson-Percival et al., 2017). In addition, LTβR agonistic antibodies, which binds LTα1β1 to induce HEVs, have been shown to boost the efficacy of anti-VEGFR2 and anti-PD-L1 combination therapy in a recalcitrant glioblastoma model (Allen et al., 2017; Schumacher and Thommen, 2022). Targeting LIGHT directly to tumor vasculature with vascular targeting peptides (VTP) induced HEVs in various tumors, improved response to ICB, and facilitated lymphocyte infiltration (Johansson-Percival et al., 2017; He et al., 2018; He et al., 2020). Intriguingly, this study has shown the depletion of Treg cells could drive HEV formation (Colbeck et al., 2017). Therapeutic induction of HEVs with ACT immunotherapy promotes lymphocyte trafficking and enhances anti-tumor response, which is a promising strategy (Lucas and Girard, 2021).

## Effector phase

Activated T cells recognize tumor cancer antigens on tumor cells by T-cell receptor (TCR) and release effector molecules to eliminate tumor cells. In TME, immune cells and tumor cells secrete and express various molecules to regulate tumor progression and metastasis. Herein, we discussed how immune cells affected tumor progression.

### T cells

According to their phenotypes, T cells are primarily classified into CD8+T cells and CD4+T cells. They play significant roles in tumor immunotherapy by releasing a variety of molecules to hamper tumor growth.

### CD8+ T cells

When stimulated by tumor-specific antigen, CD8 + T cells can secrete perforin and granzyme which can directly kill tumor cells, or mediate the apoptosis of tumor cells by the Fas/FasL signaling pathway (Hamann et al., 1997). After the initial antigen stimulation is removed, CD8 + T cells can generate a series of memory subsets under physiological conditions. Memory T- cells are divided into four categories: T memory stem cell-like (TSCM) (Gattinoni et al., 2017), central memory T (TCM), effector memory T (TEM), and tissue-resident memory (TRM) (Sallusto et al., 1999; Schenkel and Masopust, 2014). TSCM cells mostly localize in the lymph nodes and have the capacity for self-renewal. TCM cells can express the lymph node homing molecules such as CCR7 and CD62L. TEM cells produce integrins and chemokine receptors and traffic them into various tissues (Masopust et al., 2001; Sallusto et al., 2004). TCM cells and TEM cells could trigger immune activity in different tissues, but TRM cells provide a more advanced immune response (Yang and Kallies, 2021). In a mouse model, the finding suggested that TRM cell deficiency resulted in uncontrolled tumor growth with no change in the number of CD8 effector cells. Researchers further found that their anti-tumor capacity enhanced from 40% to more than 80% by increasing the number of TRM cells in TME (Nizard et al., 2017). Consequently, TRM cells are focused on gradually.

### CD8+ TRM cells

CD8+ TRM cells were initially defined in infected tissues such as the skin, lung, and intestine (Gebhardt et al., 2009; Masopust et al., 2010; Purwar et al., 2011). Gradually, CD8+ TRM cells were found in TME and were associated with the prognosis of tumor patients (Edwards et al., 2018; Savas et al., 2018; Abdeljaoued et al., 2022; Anadon et al., 2022; Jin et al., 2022; Smith, 2022). Different phenotypes are expressed by CD8+ TRM cells to destroy tumor cells effectively. First, CD103 is a characteristic marker for CD8+ TRM cells (Okla et al., 2021). CD103+ TRM-like cells possess a cytotoxic characteristic and secrete inflammatory cytokines such as GZMB, TNF-α, IL-2, and IFN-γ (Ganesan et al., 2017). They could also combine with E-cadherin on the surface of tumor cells to retain TRM in the tissue (Zhang and Bevan, 2013; Ganesan et al., 2017; Gauthier et al., 2017; Hoffmann and Schon, 2021). The expression of CD103 is highly heterogeneous. For instance, CD103 is essential in the skin, lung, and intestine (Gebhardt et al., 2009; Ganesan et al., 2017; Dumauthioz et al., 2018), but it is dispensable for the liver (Ghilas et al., 2020). CD103+ CD8+ TRM cells were associated with improved survival in cancer patients (Edwards et al., 2018; Savas et al., 2018; Hewavisenti et al., 2020; Shen et al., 2021; Huang et al., 2022b; Jin et al., 2022). For example, CD103+ CD8+ TRM cells infiltrating into TME were associated with a better adjuvant therapeutic benefit and were considered as an ideal prognostic biomarker in muscle-invasive bladder cancer. Second, CD8+ TRM cells are anchored in the tumor lesions by CD49a (VLA-1 ), which binds to collagen in the extracellular matrix (Roberts et al., 1999; Cheuk et al., 2017). When anti-VLA-1 antibodies were applied to treat patients with tumors, the number of TRM cells declined in TME (Sandoval et al., 2013). CD49a+CD8+ TRM cells produce IFN-γ to inhibit tumor progression in a melanoma mouse model, and alleviate inflammatory diseases (Cheuk et al., 2017; Le Floc’h et al., 2007; Murray et al., 2016). Moreover, CD49a also enhances the frequency of antigen encounters (Bromley et al., 2020). Third, CD69, a C-type lectin, effectively limits CD8+ TRM cell circulation by reducing the expression of sphingosine-1 phosphate receptor-1 (S1PR1), which facilitates the migration of TRM cells (Mackay et al., 1950; Bankovich et al., 2010; Skon et al., 2013). By the way, CD69 once was presumed as a marker of TRM cells, but CD69− TRM cells have also been reported (Steinert et al., 2015). CD8+ TRM cells also express chemokines like CXCR6, which promotes cell retention in the tumor sites and unleash effector functions in ovarian cancer (Muthuswamy et al., 2021a; Muthuswamy et al., 2021b). Interestingly, the level of TGF-β in TEM is required for the expression of CD103 and CD49a on the surface of CD8+ TRM cells in the lung, skin, and intestine (Zhang and Bevan, 2013; Boutet et al., 2016; Nath et al., 2019; Qiu et al., 2021a; Barros et al., 2022). TGF-β also inhibits the expression of S1PR1 through downregulating the transcription factor Krüppel-like factor 2 (KLF2) (Skon et al., 2013). Moreover, the heterogeneity of TRM cells depends on the regulation of TGF-β signaling. These findings suggested that TGF-β signaling might impact the production of TRM cells and the cytotoxicity of CD8+T cells (Mackay et al., 2013; Christo et al., 2021; Yang and Kallies, 2021). However, it is well known that TGF-β is a typical inhibitory cytokine to suppress the anti-tumor immune response. Thus, more research into the TGF-β signal pathway is required (Qiu et al., 2021b). In addition, CD8+ TRM cells express various immune checkpoint proteins, such as CTLA-4, PD-1, and PD-L1. These molecules are linked to CD8+T cell exhaustion (Gabriely et al., 2017; Philip and Schietinger, 2022). CD39 on the surface of CD8+ TRM cells also promotes tumor growth (Guo et al., 2022).

For heterogeneity of CD8+ TRM cells, researchers hypothesized several models of its differentiation, which included a separate lineage, self-maintenance, “one cell, one fate,”, and “one cell, multiple fates”. However, a plethora of studies have manifested that phenotypes of CD8+ TRM cells were specific to different tumor types, and CD8+ TRM cells were regarded as tissue-tailored (Amsen et al., 2018; Enamorado et al., 2018; Okla et al., 2021; Konjar et al., 2022). Furthermore, phenotypes of CD8+ TRM cells are inconsistent between lung cancers and healthy lung tissues (Marceaux et al., 2021). These findings have a significant impact on immunotherapy for various tumors. We also concluded the function of different phenotypes of CD8+ TRM cells (Table1). Of note, although TRM cells play a crucial role in autoimmune diseases and viral infections, they are still in infancy in human tumors.

**TABLE 1:** The rRole of TRM cells in cancer patients.

Cancer types	Phenotype	The function of TRM cell	References
Lung cancer	CD103, and CD8	High CD103+ CD8+ TRM tumor infiltration boosts anti-tumor activity	Tarantino et al. (2022)
	CD103, and CD8	CD8+TRM tumor infiltration reduces the risk of metastasis	Mehdizadeh et al. (2021)
	CD103, and CD8	CD8+TRM in TLS prolongs the survival (*p* < 0.05)	Martinez and Moon, (2019)
	CD8, CD103, CD69, and CD49a	CD8+ TRM cell infiltration is positively associated with a better prognosis	Burnet. (1970); Dunn et al. (2002); Whiteside. (2012); Wilczyński and Nowak. (2012); Duhan and Smyth. (2021); Wada et al. (2022)
Melanoma	CD69, CD8, and CXCR6	Tumor-specific TRMs have a role in limiting the invasion of the tumor into the other tissues	Munari et al. (2021)
	CD39, CD103, and PD-1	High CD39+ TRM infiltration is associated with a better outcome	Nagarsheth et al. (2017)
	CD8, CD103, and CD69	CD8+ TRMs enhance anti-tumor response	Lamplugh and Fan, (2021)
	CD8, CD103, CD69, CD49a, PD-1, and LAG-3	A high proportion of CD8+ TRMs are positively associated with the clinical outcome	Davis et al. (2015); Goossens et al. (2019); Petty et al. (2019)
Bladder cancer	CD103, and CD8	High-level CD103+CD8+TRM cell infiltration enhances the efficacy of immunotherapy	Eryn and Ott. (2021); Tarantino et al. (2022)
	CD103, and CD8	TRM cells infiltrating the tumors are linked to lower tumor stage	Lu et al. (2014)
	CD103, CD8, CD69, and CD49a	The high density of CD8+ TRMs is positively associated with a good prognosis	Kvedaraite and Ginhoux, (2022)
Ovarian cancer	CD3, CD8 CD103, and CD69	CD103+ CD8+ TRMs in tumor site enhance anti-tumor immunity	Bogunovic et al. (2009); Böttcher et al. (2018)
	CD103, CD8, PD-1, and CD3	High proportions of CD8+ TRMs have a positive correlation with the prognosis	Roberts et al. (2016); Villani et al. (2017); Barry et al. (2018)
Breast cancer	CD103, and CD8	CD8+ TRM infiltration reduces the release rate (RFS; *p* = 0.002)	Matsuo et al. (2018)
	CD8, CD103, CD69, and PD-1	CD8+CD103+ TRM infiltration is associated with a favorable prognosis	(Sánchez-Paulete et al., 2018; Cancel et al., 2019; Ferris et al., 2020)
Pancreatic ductal adenocarcinoma	CD8, and CD103 PD-1	Increased numbers of CD8+ TRMs are associated a better prognosis (DFS: *p* = 0.22, OS: *p* = 0.009)	Schoenberger et al. (1998)
Liver cancer	CD8, and CD103	The number of CD8+ TRMs is positively correlated with the prognosis (OS: *p* < 0.0001)	de Mingo Pulido et al. (2018)
Gastric cancer	CD8, and CD103	Low levels of CD8+ CD103+ TRM cells are associated with a worse prognosis	Chow et al. (2019)
	CD103, CD69, PD-1, TIGIT, and CD39	CD8+ TRMs amplify anti-tumor response	Stratikos et al. (2014)
cutaneous squamous cell carcinoma	CD8, and CD103	CD8+ CD103+ TRM cells are negatively associated with OS	Garris et al. (2018)
Head and neck cancer	CD8, and CD103	High CD103+ cell infiltration is associated with a good prognosis (OS: *p* = 0.0014, DSS: *p* = 0.0015, DFS: *p* = 0.0018)	Mikucki et al. (2015)

DFS, disease-free survival; DSS, disease-specific survival; OS, overall survival; RFS, relapse-free survival.

### The CD8+ TRM-based targeted therapies

According to the known functions of TRM cells, researchers have proposed some approaches to fortify the function of TRM cells and enhance anti-tumor response. First, the treatment with PD-1 inhibitors enhanced the capacity of CD8+ TRM cells in melanoma, lung cancer, and esophageal cancer (Edwards et al., 2018; Han et al., 2020; Abdeljaoued et al., 2022). Furthermore, in the preclinical melanoma model, using the combination of CD39 inhibitor and ICB made tumor growth retardation (Sade-Feldman et al., 2018). Recently, a bispecific CD28H/PD-L1 antibody has been developed, which could increase the number of TRM cells and enhance anti-tumor immunity (Ramaswamy et al., 2022). Second, vaccines have been designed to treat tumors. In a preclinical cervical cancer model, the HPV vaccine promoted CD103 expression on the surface of TRM cells and effectively prolonged the survival (Sandoval et al., 2013; Komdeur et al., 2017). By the same token, using STxB-E7 vaccination enhanced the number of TRM cells and delayed tumor growth in HNSCC (Mondini et al., 2015). After using Polypoly-ICLC-assisted tumor lysate vaccine to treat patients with low-grade gliomas, the drugs acquired a good efficacy and the number of CD8+ TRM cells increased in TME (NCT02549833). Treatment with cervicovaginal vaccination with HPV16 E7aa4362 peptide/CPG-1826 could induce the production of CD103+ CD8+ TRM cells, and; subsequently, the number of CD8+ T cells increased, resulting in suppressing tumor progression in the genital tract (Huang et al., 2022b). Vaccines were also applied to generate TRM cells in mouse models of various infections (Zens et al., 2016; Yang and Kallies, 2021; Zheng and Wakim, 2021). Researchers also attempted to utilize ACT to hinder tumor growth (Lim and June 2017). Using the adoptive transfer of expanded CXCR6+ TRM cells has acquired the benefits in gastrointestinal cancer (Abdeljaoued et al., 2022). Reprogramming DCs to induce CD103 expression of CD8+ TRM cells has acquired obvious efficacy in a preclinical model of breast cancer (Wu et al., 2014). In a melanoma mouse model, short-term depletion of CD11c+ cells not only facilitated TRM cell trafficking but also was favorable for long-term TRM cell maintenance (Vella et al., 2021). Of note, few clinical trials have been performed to dissect TRM cell functions in different tumors (Craig et al., 2020). In a nutshell, CD8+ TRM cells potentially serve as a critical role, but some challenges remain. For instance, what are the mechanisms by which TRM cells enhance anti-tumor immunity? Are the phenotypes consistent between normal tissues and tumor cells? Which phenotypes could define TRM? How are the TRM cells maintained and replenished in TME? Therefore, these problems will trigger intense research.

### CD4+ T cells

CD4+ T cells play a pivotal role in mediating adaptive immunity by various mechanisms. Over the past decades, extensive research suggested that CD4 +T cells could be mainly divided into T-helper 1 (Th1) cells, T-helper 2 (Th2) cells, T-helper 17 (Th17) cells, follicular helper T cells, and regulatory T cells. Th1 cells secrete IL-2 and IFN-γ. IL-2 promotes CD8+ T cell proliferation and activation, as well as the development of CD8+ memory cells (Kim et al., 2006; Williams et al., 2006). IFN-γ facilitates the process of antigen presentation (Dong, 2021). Th2 cells produce IL-4, IL-5, and IL-10 to exert their function. For example, after pathogens have been cleared, IL-10 inhibits innate immunity and function of Th1 cells, which could maintain host immune homeostasis (Couper et al., 1950). Th17 cells principally facilitate the death of extracellular bacteria and fungi (Luckheeram et al., 2012). Because of the complicated function of Tfh cells and Treg cells in anti-tumor immunity, thus, we mainly discussed the roles of Tfh cells and Treg cells.

### Follicular helper T cells

Tfh cells, accumulated in the GCs of SLO and TLS, express a variety of phenotypes which are essential for the formation and maturation of the GCs (Asrir et al., 2017; Ribeiro et al., 2022; Schmidleithner and Feuerer, 2022) and improve the prognosis in breast cancer, colorectal cancer, and pancreatic ductal adenocarcinoma (PDAC) (Yamaguchi et al., 2020; Lin et al., 2021; Noël et al., 2021). IL-21 secreted by Tfh cells activates the STAT3 signaling pathway to induce the expression of transcription factor B cell lymphoma 6 (BCL6) and participate in the differentiation of Tfh cells (Nurieva et al., 2008; Linterman et al., 2010; Lüthje et al., 2012). IL-21 also plays a pivotal role in triggering CD8+T cell function and tumor regression in the lung adenocarcinoma model (Cui et al., 2021). BCL6 is the main transcription factor which upregulates the expression of CD28 and CXCR5, promotes the differentiation of Tfh cells through repressing Blimp1, and is important for GC to respond to tumor antigens (Nurieva et al., 2009; Yu et al., 2009; Ciucci et al., 2022). Of note, CD28 is required for the differentiation of Tfh cells. If CD28 was deficient in T cells or reduced by its inhibitor, the differentiation of Tfh cells was blocked. Tfh cells could express CTLA-4, which binds to CD80/CD86 and leads to the inhibition of CD28 (Hart and Laufer, 2022). Tfh cells are recruited into the B cell zone to form GC by expressing CXCR5, which combines with CXCL13 + B cells (Kim et al., 2001). CD40L on the surface of Tfh cells activates B cells and sustains the survival of GC B cells by binding CD40 (Vinuesa et al., 2016). Tfh cells also express ICOS. ICOS binding to its ligand ICOSL is essential for the survival of GC B cells and the maintenance of Tfh cell phenotypes by reducing the Kruppel-like factor 2 (Liu et al., 2015; Weber et al., 2015). In addition, other cytokines have different roles to affect Tfh functions. High-level IL-2 secreted by Th1 cells mediates the impairment of Tfh function through activating STAT5 signaling, whereas IL-6 secreted by DCs inversely prevents STAT5 from the combination of the IL-2rb locus (Hart and Laufer, 2022). Astoundingly, TGF-β in humans plays a protective role for Tfh cells, which activates STAT3 and STAT4 by interacting with IL-12 and IL-23, and silences genomic organizer SATB1 to aid Tfh cell differentiation (Kurata et al., 2021; Chaurio et al., 2022; Schmidleithner and Feuerer, 2022). However, it is a negative regulator in mice, and using TGF-β inhibitors reduces Tfh accumulation in the tumor sites (McCarron and Marie, 2014; Niogret et al., 2021). Although Tfh cells have been explored, it is deficient for the mechanism of Tfh differentiation and the function of GCs in various tumors.

### Tfh-related immunotherapies

Recently, studies have found that the presence of Tfh cells is important for upregulating CD8-dependent anti-tumor immunity and improving the benefit of anti-PD-L1 therapy in tumors (Chen et al., 2021; Niogret et al., 2021). Immune checkpoint inhibitors also facilitated Tfh cells to activate B cells and further improved the anti-tumor response in specific breast models (Hollern et al., 2019). In addition, anti-CXCR5 CAR-T cells were applied to treat B cell Nonnon-Hodgkin’s lymphoma (B-NHLs), which eliminated B-NHL cells and lymphoma-supportive Tfh cells (Bunse et al., 2021). In a study, targeting Bcl6 – Blimp1 axis has been proposed to facilitate T cell differentiation, but the drug has not been generated (Ciucci et al., 2022). These data provide a treatment strategy for Tfh cells, but it is required to further investigation for the role of Tfh cells in human tumors.

### Treg cells

Treg cells, another subset of CD4+ T cells, are responsible for immunosuppression and help tumor cells avoid immune surveillance. Tregs can be divided into three populations: naïve Tregs (FOXP3low, CD25low, and CD45RA+), eTregs (FOXP3high, CD25high, and CD45RA-), non-Tregs (FOXP3low, CD25low, and CD45RA-) based on themselves their phenotypes. The eTreg acts as a vigorous suppressor, whereas non-Tregs are immunostimulatory and secrete IFN-γ (Miyara et al., 2009). Emerging evidence indicated that eTregs resulted in a poor prognosis, but non-Tregs infiltration in colorectal cancer (CRC) was associated with a favorable outcome (Saito et al., 2016). Thus, a challenge was posed that distinguished the types of FOXP3+ Tregs in tumors (Kim et al., 2020). Further analysis found that the prognostic value of intratumoral Tregs in various tumors is inconsistent (Shan et al., 2022). In order to identify Tregs and dissect their functions, we must understand the phenotypes and cytokines expressed by Tregs. FOXP3 is a credible marker of Treg cells, and is essential for maintaining the function of Treg cells. It is reported that loss of FOXP3 expression could impair the stability of Tregs and transform Tregs into effector cells (Qu et al., 2022). It is intriguing that CD25 binding to IL-2 could activate STAT5 signaling and then induce expression of FOXP3 to inhibit CD8+ T cell response (Chinen et al., 2016). A study has also shown that CD45RA + Tregs play a suppressive role and are associated with an unfavorable prognosis in CRC (Saito et al., 2016). Cytokines secreted by Tregs, such as IL-10, IL-35, and TGF-β, are key factors in inhibiting the function of NK cells and effector T cells and promoting tumor progression (Qu et al., 2022). Increased IL-10 and IL-35 have been associated with worse outcomes in cancer patients (Zhao et al., 2015; Turnis et al., 2016). IL-35 also elicits the expression of inhibitory molecules on Teffs like TIM-3 and CTLA-4, which induces Teffs into the exhaustion status (Turnis et al., 2016; Sawant et al., 2019). IL-10 impairs CD8+ T cell function, and inhibits the expression of MHC II molecules and APCs activation (Wang et al., 2019). TGF-β is a crucial mediator for immunosuppression in the TME, which fosters the expression of FOXP3 on Tregs (Turnis et al., 2016; Colak and Ten Dijke, 2017), and induces the conversion of Th17 cells into Tregs, resulting in immune tolerance (Gagliani et al., 2015). Notably, Tregs could release GZMB and perforin to directly kill effector T cells and NK cells in TME (Cao et al., 2007). Furthermore, antigen-specific Tregs could disturb the combination of the effector T cells and cognate antigen by interacting with APC (Qu et al., 2022). Tregs also express CD39 and CD73, resulting in adenosine aggregation in TME (Allard et al., 2020). Additionally, CCR4 is the most studied receptor that can recruit Tregs into TME and promote tumor growth by binding to CCL22 or CCL17 (Gobert et al., 2009). Tregs express immune checkpoint molecules to bolster their function, such as TIM-3 and CTLA-4 (Dixon et al., 2021).

### Treg-based targeted therapies

Based on these immunosuppressive mechanisms of Tregs, researchers have proposed numerous noteworthy therapeutic strategies. First, the depletion of Tregs *via* anti-CD25 mAb (daclizumab) and toxin conjugated anti-IL-2 (denileukin diftitox) induced tumor regression and prolonged disease-free survival (DFS) in tumors (Solomon et al., 2020; Nishikawa and Koyama, 2021). Despite the fact that anti-CD25 mAb could deplete Tregs in melanoma, it did not elicit an anti-tumor immune response (Luke et al., 2016). The anti-CD25 antibody, RG6292, designed to deplete Tregs without disturbing IL-2 signaling on effector T cells, has been applied in a mouse model (Solomon et al., 2020) and is currently being tested in human tumors (NCT04158583). Furthermore, immune checkpoint inhibitors (ICIs) like anti-CTLA-4 antibody or anti-TIGIT antibody combined with the blockade of CD25 potently resulted in the depletion of Tregs and enhanced anti-tumor responses in a mouse model (Arce Vargas et al., 2017). Near-infrared photoimmunotherapy (NIR) was also used to precisely deplete Tregs in TME (Sato et al., 2016). Second, It has been reported that using AZD8701, which targets FOXP3 on Tregs, reduces the number of FOXP3 expression in mouse models (Sinclair et al., 2019), and its clinical trial is ongoing (NCT04504669). Epigenetic modifiers have been designed to target genes that regulates FOXP3 expression on Tregs, leading to the depletion of Tregs. For instance, targeting Treg-specific demethylated region (TSDR) and histone deacetylation reduced FOXP3 expression on Tregs (Ma et al., 2018; Nagai et al., 2019). Third, CCR4 blockade may reduce the accumulation of Tregs in the tumor sites and improve therapeutic benefits in different types of cancers. Mogamulizumab, a defucosylated anti-CCR4 mAb, has been approved to treat patients with Sézary syndrome, a cutaneous T cell lymphoma. It has been tested for the clinical response in phase 1 clinical trials in various solid tumors (Shan et al., 2022). FLX475, another CCR4 inhibitor, is currently being evaluated alone or in combination with anti-PD-1 and anti-CTLA-4 for the treatment of advanced tumors (Shan et al., 2022). TNFR2-expressing Tregs play a potently immunosuppressive role in human tumors, so targeting TNFR2 has been generated such as APX601, which is tested and resulted in reducing Treg frequency and hindering Treg function in tumors (Hariyanto et al., 2022). Moreover, TGF-β receptor inhibitors have been investigated. TGF-β-R inhibitors (Galunisertib) suppress Treg function and control tumor growth (Holmgaard et al., 2018). The combination therapy of galunisertib and ICIs further reduced Treg numbers in a mouse melanoma model, and this approach is being investigated in human tumor (Ravi et al., 2018). Glycoprotein-A repetitions predominant (GARP) could facilitate the secretion of TGF-β and Treg function in preclinical models. Using the anti-GARP antibody, S1055a, could lead to the depletion of Tregs and activate effector T cells in preclinical models, and this drug is being investigated in a clinical trial (Shan et al., 2022). Besides, TGF-β-responsive CAR-T cells could prevent naïve T cells from differentiating into Tregs and promote anti-tumor immunity (Zhang et al., 1950). DC/4T1Adv-TGF-β-R fusion vaccine could inhibit tumor-derived TGF-β, which leads to the reduction of Tregs and favor anti-tumor immunity in the mouse model (Hou et al., 2018). In HPV positive cancers, a clinical trial, treatment with HPV vaccination alone or in combination with anti-PD-L1/TGF-β Trap (M7824), is underway (NCT04432597). Another clinical trial, using a TGF-β receptor ectodomain-IgG Fc fusion protein inhibitor of TGF-β in solid tumors, also is being investigated (NCT03834662). In brief, targeting T subsets is important for cancer immunotherapy. Despite enormous progress in the field, a further analysis needs to be conducted.

### Tertiary lymphoid structures

Tertiary lymphoid structures which have been already mentioned are defined by an inner B-cell zone and an outer T-cell zone. B cells are indispensable for TLSs. Currently, activation of B cells in infections and autoimmune diseases has been studied, but little research has been performed in different cancers (Cogné et al., 2022). Naïve B cells could be activated through the interactions between BCR and tumor antigens, upon activated CD40 signaling (Cancro and Tomayko, 2021). In PDAC, immature B cells present in TLS only express IgD, and mature B cells express IgG and IgM (Andrew et al., 2021)). Likewise, in lung cancer, naive B cells express IgD, but mature B cells express IgD-CD38+ CD138+ (plasma cell) (Germain et al., 2014). These findings indicate B cell activation maybe undergo class-switch recombination (CSR). The activated induced deaminase (AID) expressed by B cells is required for CSR and could promote somatic hypermutation (SHM) (Dieu-Nosjean et al., 2016; Lehmann-Horn et al., 2016). Isotype class switching depends on different cytokines released by Tfh cells. For instance, upon the presence of IFN-γ in GC, IgG2a and IgG3 were expressed by B cells, but IgG2a and IgG3 were also converted to IgE mediated by IL-4 (Kinker et al., 2021). IgG and IgA antibodies secreted by plasma cells could recognize tumor antigens and control tumor cell growth. It is reported that high-level IgG antibody *in vitro* was correlated with a worse prognosis in breast cancer patients, but IgA antibody *in vitro* that reacts to tumor antigens is associated with TLS presence in TME (Garaud et al., 2018). In another study, a high-level IgG antibody is associated with a better immune response. Moreover, supernatants (SNs), including IgG and IgA antibodies, were used to evaluate the immune responses to 33 tumor antigens, and the results were different (Germain et al., 2014). Thus, the role of antibodies produced by GC B cells must be further explored. For the TLS formation, it is currently being explored, but researchers have demonstrated that the combination of 5-Aminoleuvulinic aminoleuvulinic acid-photodynamic therapy (ALA-PDT) and anti-PD-L1 mAb could promote the TLS formation and then enhance the clinical outcome in cutaneous squamous cell carcinoma (Zeng et al., 2022). Another study has also reported that TGFB1 mRNA expression was also associated with TLS formation in ccRCC (Takahara et al., 2022). However, the research has shown that tumor-associated sensory neurons are negatively correlated with mature tertiary lymphoid-like structures and HEVs (Vats et al., 2022).

**FIGURE 1:**
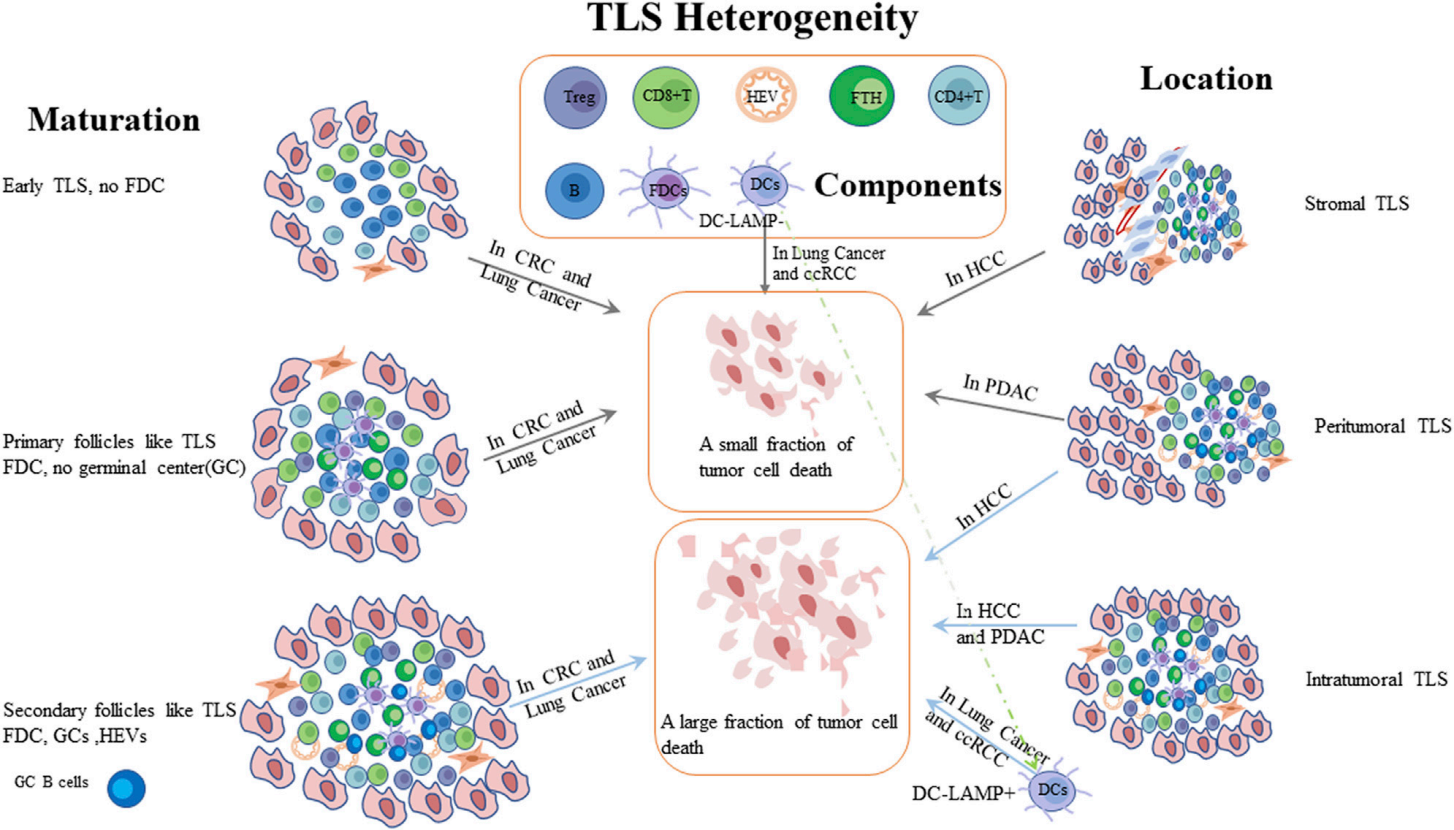
Patients with cancer have different prognosis due to TLS heterogeneity. Compared to stromal TLS, intratumoral or both intratumoral and peritumoral mature TLSs were associated with a better prognosis in different tumors. TLSs with GCs have been shown to kill tumor cells more effectively than immature TLS. PDAC: pancreatic ductal adenocarcinoma; HCC: hepatocellular carcinoma; CRC: colorectal cancer; ccRCC: clear cell renal cell carcinomas; DC-LAMP: Dendritic dendritic Cell cell Lysosomelysosome-–Associated associated Membrane membrane Proteinprotein; FDC: follicular dendritic cells; HEV: high endothelial venules.

Growing evidence showed that TLSs were associated with clinical outcomes of cancer patients (Schumacher and Thommen, 2022). Although TLSs were frequently correlated with a favorable prognosis in human tumors, but some studied have reported that TLSs were also linked to a negative correlation with clinical outcomes in hepatocellular carcinoma (HCC) and clear-cell renal carcinoma (ccRCC) (Finkin et al., 2015; Jacquelot et al., 2021a) or no impact on OS in melanoma and prostate cancer (Ladányi et al., 2014; García-Hernández et al., 2017). Moreover, the prognostic value of TLSs is inconsistent with the same tumor types, such as HCC and breast cancer (Liu et al., 2017; Calderaro et al., 2019). These inconsistencies might be explained by TLS heterogeneity, including TLS maturation state, location or detected phenotypes in tumors (Jacquelot et al., 2021a) (Figure 1). With respect to TLS location, the prognostic values differ from tumor types. The location and maturity of TLS contribute to the difference in HCC prognosis. Compared to TLS situated in stromal tumor, the intratumoral and peritumoral mature TLSs were associated with a favorable prognosis (Calderaro et al., 2019). Pancreatic cancer with intratumoral TLS signified a better prognostic value and exhibited a lower infiltration of immunosuppressive cells and higher infiltration of T and B cells compared to peritumoral TLS (Hiraoka et al., 2015). TLSs could also predict the prognosis of patients with tumor metastases. In melanoma and breast cancer, no representative phenotypes of TLS was observed in brain metastases (Cipponi et al., 2012; Lee et al., 2019). Besides, TLS density was related to primary tumor types in metastatic organs (Remark et al., 2013; Schweiger et al., 2016; Montfort et al., 2017; Lee et al., 2019). For example, TLS levels were found to be high in patients with lung metastases from colorectal and breast cancers. With regard to TLS maturation, TLS maturation were divided into three types: early, primary-, and secondary follicle–like TLS (Posch et al., 2018). The different degrees of maturation of TLS denoted inconsistent prognostic values in CRC, because early TLS without GCs had almost no impact on clinical outcome compared to mature TLS which signified a better outcome (Di Caro et al., 2014; Posch et al., 2018). In patients with lung squamous cell carcinoma, both early and primary TLSs did not affect patient survival, and only secondary TLSs exerted a favorable role in the prognosis (Siliņa et al., 2018). In preneoplastic hepatic lesions, immature TLSs did not effectively inhibit tumor cell growth (Meylan et al., 2020). Immature TLS without dendritic cell lysosome-associated membrane protein (DC-LAMP) exhibited a worse prognosis than existing TLS with DC-LAMP in NSCLC and ccRCC (Giraldo et al., 2015) 243). However, whether TLS is mature or not, its presence is associated with positive outcomes in oral squamous cell carcinoma (Li et al., 2020a). Remarkably, the most important factor should be the components of TLSs in various tumors. Tfh cells and B cells could express various chemokines to promote TLS formation. The presence of HEVs aids immune cells migration. These components have the potential to improve clinical outcomes. However, Tregs, the component of TLSs, play an immunosuppressive role and result in tumor growth (Martinet et al., 2012; Gu-Trantien et al., 2017; Ishigami et al., 2019). As a side note, a study has supposed that follicular Treg (Tfr) cells might be a key factor to reduce the number of CD8+T cells in adenocarcinoma (Wang et al., 2022). Moreover, it was reported that the plasma cells are crucial for the efficacy of ICB in the presence of TLS, but the molecular and cellular mechanisms for promoting plasma cells to response ICB are still unclear. Thus, the role of plasma cells in presence of TLS needs to be further explored (Teillaud and Dieu-Nosjean, 2022). TLS with high levels of M2 macrophages and CD4+THC cells (CD3+CD8−Bcl6− ) correlates with tumor progression and a higher recurrence rate in patients with CRC (Yamaguchi et al., 2020). In NSCLC, the subgroup with low-level DC-LAMP + DCs and high-level CD8+T cells reduced the likelihood of survival, suggesting the importance of DC-LAMP + DCs in TLS (Goc et al., 2014). Noteworthily, researchers have also reported that TRM could promote TLS maturation, and the number of TRM was more abundant in mature TLS in patients with lung adenocarcinoma. Furthermore, high-level TRM within TLS, especially CD103+ TRM, was associated with a better prognosis (Yang et al., 2022; Zhao et al., 2022). However, the components still need to be explored in the future. Some studies also proposed that the density of TLSs varied at different stages of the tumor. TLSs were less abundant in T3 and T4 stages compared to T1 and T2 stages of oral squamous cell carcinoma, but TLSs were more abundant in advanced stages (II-IV) than in stage I gastric cancer and high-grade breast cancer (Sautès-Fridman et al., 2019). Another study also reported that the number of TLS might be associated with the prognosis and could be considered as a target for treating patients with urachal carcinoma (Zhang et al., 2022a). Based on these conclusions, it is urgent to precisely understand the formation, components, and mechanism of TLS. Researchers have hypothesized the formation and maturation process of TLS in CRC and NSCLC, respectively, but there is a lack of evidence to support it (Meng et al., 2021). Hence, a comprehensive analysis of TLS is an area of immense interest.

Inducing or improving TLS function not only enhances anti-tumor responses, but also promotes the expansion of autoreactive T and B cells. First, the presence of intratumoral TLS has been regarded as a favorable marker of the responsiveness of ICB therapy in lung cancer, ccRCC, bladder cancer, urothelial carcinoma, melanoma and soft-tissue sarcoma (Groeneveld et al., 1990; Petitprez et al., 2020; van Dijk et al., 2020; Voabil et al., 2021). Accordingly, ICB increased the density of TLS or induced TLS formation in the tumor sites (Rita et al., 2020). Besides, ICB therapy combined with CXCL13 facilitated immune cell infiltration and TLS formation (Hsieh et al., 2022). Second, therapeutic vaccination also induced TLS formation in specific tumors. For instance, therapeutic vaccination targeting HPV16 and HPV18 induced TLS formation compared to non-vaccinated patients in high-grade cervical intraepithelial neoplasia (CIN2/3) (Maldonado et al., 2014). In PDAC, a specific vaccine, an irradiated, allogeneic granulocyte–macrophage colony-stimulating factor–secreting pancreatic tumor vaccine (GVAX), in combination with cyclophosphamide, was used to elicit TLS formation *via* suppressing the Treg pathway and activating the Th17 cell pathway. Lastly, the induction of HEV has already been elaborated (Lutz et al., 2014). To sum up, the role of TLS has been stated above. Thus, it is worthy of a comprehensive investigation of TLS, including the formation of TLS, the mechanisms of controlling tumor progression, and the interactions of TLS and immunotherapies, even the strategies for targeting TLS in TME.

### Innate lymphoid cells

Innate lymphoid cells (ILCs) are an important part of the immune system to defend against tumor cells on the front line. ILCs are divided into five categories on the basis of cytokines and specific transcription factors, including natural killer (NK) cells, lymphoid tissue inducers, helper ILC1s, helper ILC2s, and helper ILC3s (Spits et al., 2013; Vivier et al., 2018). These cells, which lacks antigen-specific receptors, have different functions through secreting cytokines or activating specific signaling pathways.

### Natural killer cells

NK cells have the potential to mediate anti-tumor immunity *via* directly or indirectly killing tumor cells. NK cells can be defined by the expression of CD16 and CD56 markers, but somatically rearranged antigen receptors like TCR is scarce (Myers and Miller, 2021; Stefania et al., 2021). Accordingly, NK cells are categorized into two subsets: CD56brightCD16- and CD56dimCD16+ NK cells. CD56bright NK cells not only release a variety of cytokines, but also interact with various molecules secreted by other immune cells (Fehniger et al., 1950; Cooper et al., 2001; Wagner et al., 2017a). CD56dimCD16+ NK cells rapidly mediate antibody-dependent cellular cytotoxicity (ADCC) through secreting granzyme and perforin (Bryceson et al., 2006; Stabile et al., 2015; Voskoboinik et al., 2015; Freud et al., 2017; Bald et al., 2019; Prager et al., 2019). With respect to the cytotoxicity of NK cells, the cytotoxicity receptors exert a powerful influence, including CD16 and the natural cytotoxicity receptor family, such as NKp30, NKp40, NKp44, and NKp46. CD16 is the strongest activating receptor and a trigger to ADCC without the assistance of other receptors (Bournazos et al., 2017). The natural cytotoxicity receptor family combined with tumor-associated ligands to remove malignant cells (Kruse et al., 2014; Barrow et al., 2019; Karagiannis and Kim, 2021). NKG2D is another important activating receptor, which recognizes MHC class I chain–related proteins sequence A (MICA) and MICB and then promotes the production of IFN-γ (Zompi et al., 2003; Raulet et al., 2013). NKG2D also interacts with transmembrane adaptor protein DAP10 to enhance the cell cytotoxicity (Sivori et al., 2021). Of note, soluble NKG2D ligands released by tumor cells have been reported to correlate with poor outcomes (Lanier, 2015; Ferrari de Andrade et al., 2018). Likewise, soluble NKp30 ligand from tumor cells promoted tumor progression and metastasis (Semeraro et al., 2015). On the surface of NK cells, inhibitory receptors also are expressed, which contains immunoreceptor tyrosine-based inhibitory motifs (ITIMs) (Myers and Miller, 2021). The inhibitory KIRs (iKIRs) recognize and bind to class I HLA molecules to hinder activating signals and impair NK cell functions (Guillerey et al., 2016; Chiossone et al., 2018). NKG2A/CD94 heterodimers combine with HLA-E molecules to impede their cytolytic activity and might assist tumor cells to evade immune surveillance. NKG2C/CD94 heterodimers, on the other hand, activate NK cells by binding to HLA-E, and their activation is dependent on NKG2A (Shifrin et al., 2014; Sivori et al., 2019; Myers and Miller, 2021). As a side note, the KIRs have both activating and inhibitory functions (Sivori et al., 2021). As mentioned previously, NK cells promote anti-tumor immunity through releasing IFN-γ, TNF-α, granzymes and perforins, but they could transdifferentiate into helper ILC1s (hILC1s ) under activated TGF-β signaling, resulting in impairing NK cell-mediated tumor control (Cortez et al., 2017; Gao et al., 2017; Cuff et al., 2019; Jacquelot et al., 2022). Besides, IL-15 signaling also triggers NK cells to convert into hILC1-like cells in head and neck cancer, but whether hILC1 cells can differentiate into NK cells is still unclear (Jacquelot et al., 2022). NK cell cytotoxicity was associated with clinical outcomes of cancer patients. Some studies have demonstrated an enhanced prognosis with tumor‐associated NK cells in CRC (Tartter et al., 1960), renal cancer (Eckl et al., 2012; Chiossone et al., 2018), melanoma (Messaoudene et al., 2016; Cursons et al., 2019), gastric cancer (Du and Wei, 2018), and HCC (Zhang et al., 2017a). However, NK cell infiltration exerts a negative influence on the prognosis in NSCLC (Platonova et al., 2011), breast cancer (Mamessier et al., 2011; Liu et al., 2021a), and renal cell carcinoma (Schleypen et al., 2003). These paradoxical observations are mainly based on the level expression of receptors or production of functional molecules.

### Helper ILC1

Both hILC1s and NK cells express secrete IFN-γ, TNF-α, and transcription factor T-bet, but hILC1s do not depend on Eomes and have lower cytotoxicity (Bernink et al., 2013; Kim et al., 2021). Based on these features, NK cells and hILC1s mirror CD8+T cells and CD4+T, respectively (Gordon et al., 2012). In the context of cancer, hILC1s have both a tumoricidal function and an immunosuppressive function. When the presence of TGF-β in TME, hILC1s induced the development, growth and metastasis of tumors (Tumino et al., 2020). Although the hILC1s secrete IFN-γ to kill tumor cells (Castro et al., 2018; Verma et al., 2020), the IFN-γ can drive EMT leading to carcinogenesis (Wang et al., 2020a), and tumor cells escape (Zaidi and Merlino, 2011). When the function of hILC1s was impaired, TNF-α production decreased, resulting in a pro-tumor effect in patients with tumor (de Weerdt et al., 2016; Gao et al., 2017). Several studies have shown that the presence of hILC1s has a paradoxical prognosis in various tumors (Dadi et al., 2016; Salimi et al., 2018; Qi et al., 2021). Intriguingly, one study found that hILC1s predominantly expressed activating receptors in the early stage of CRC, but they converted to expressing inhibitory receptors in the advanced stage (Qi et al., 2021).

### Helper ILC2

The hILC2s could release various cytokines and express transcription factors, including IL-4, IL-5, IL-13, IL-33 receptor, GATA3, and RORα (Entwistle et al., 2019). IL-33 is a major activator of hILC2s by binding to the IL-33 receptor. Some studies have shown that a large number of hILC2s infiltrate and exert an anti-tumor effect in IL-33 enriched the tumor sites (Kim et al., 1950; Jacquelot et al., 2021b). For instance, IL-33 activated hILC2s, which released granulocyte-–macrophage colony-stimulating factor (GM-CSF) and eosinophils were attracted to the tumor location. These activities eradicated tumor cells in melanoma (Jacquelot et al., 2021b). However, IL-33 also promotes tumor development and angiogenesis by various mechanisms (Maggi et al., 2020). For instance, IL-33 could raise the number of CD4+FOXP3+Tregs to suppress immune activity. Accordingly, hILC2s have played both pro-tumor and anti-tumor roles. The hILC2–MDSC regulatory axis has been discovered in various tumors (Chevalier et al., 2017; Trabanelli et al., 2017; Maggi et al., 2020). The hILC2s secret IL-13 to activate MDSCs which could inhibit anti-tumor immunity, and MDSCs, in turn, produce IL-13 to enhance immunosuppressive activity further (Maggi et al., 2020). Besides, the anti-tumor function of hILC2s also has been reported in HCC (Xu et al., 2021a; Heinrich et al., 2022), CRC (Ercolano et al., 2021; Huang et al., 2021; Qi et al., 2021), pancreatic cancer (Moral et al., 2020), and melanoma (Wagner et al., 2020; Peng et al., 2021a; Jacquelot et al., 2021b).

### Helper ILC3

The roles of hILC3s in cancer prognosis are controversial, which expresses IL-17, IL-22, IL-23 receptor, GM-CSF, and the RORγt (Penny et al., 2018; Meininger et al., 2020). In NSCLC, hILC3s produce IL-22, and TNF-α, recruit Teff cells, and promote TLS formation to prolong the survival (Carrega et al., 2015; Goc et al., 2016). In contrast, in breast cancer, IL-22 produced by hILC3s impelled tumor proliferation and metastasis (Irshad et al., 2017). In CRC, ILC3s produced IL-22 which activated STAT3 phosphorylation signaling to promote the development and invasion of tumor (Kirchberger et al., 2013). Additionally, IL-22 is important to maintain and repair the epithelial barrier (Goc et al., 2016; Mao et al., 2018). GM‐CSF produced by hILC3s could attract macrophages in the gut and induce the generation of FOXP3+Treg cells to counteract the immune response (Mortha et al., 2014). IL-17 released by hILC3 played a role in tumorigenesis of the liver with infection of Helicobacter hepaticus and CRC (Wang and Karin, 2015; Han et al., 2019). In human squamous cervical carcinoma and breast cancer, high-level IL-17 played a pro-tumor role (Punt et al., 2015; Irshad et al., 2017). IL-12 secreted by hILC3s inhibited tumor development in melanoma (Eisenring et al., 2010; Wu et al., 2020). In breast cancer, RORγt + hILC3s could also enhance the likelihood of lymph node metastasis (Irshad et al., 2017). Recently, a new subset of ILCs, regulatory ILCs, has been reported, which releases IL-10 following TGF-β signaling to play a tumor-promoting role (Wang et al., 2017; Bald et al., 2019; Wang et al., 2020b). High levels of IL-23 in TME binding to IL-23 receptors expressed by hILC3s were associated with gut tumorigenesis (Man, 2018; An et al., 2019). LTi cells are important components to assist the formation of Peyer’'s patches and lymphoid neogenesis and inhibit tumor growth (Tumino et al., 2020).

### The interactions of hILCs

The phenotypes and functions of hILC subsets changed under different microenvironments. For example, hILC2s converted to hILC1s by expressing the receptors for IL-1β, IL-12, and IL-18, and further expressed hILC1 phenotypes, such as T-bet, IFN-γ. Additionally, under the presence of IL-4, hILC2s were reversed (Bald et al., 2019; Salvo et al., 2020). Under the influence of cytokines like IL-12, IL-23, and IL-1β, hILC3s exhibited the characteristics of hILC1s as well as cytotoxic activity against tumor growth in melanoma (Nussbaum et al., 2017; Cella et al., 2019). In pulmonary squamous cell carcinomas (SqCC), hILC3s derived from hILC1s conversion suppressed anti-tumor immunity and thus shortened patient survival (Koh et al., 2019). Besides, in the presence of TGF-β, hILC2s were converted into hILC3-like cells and hILC3s were converted into ILCregs (Koh et al., 2019). However, the conversion masochisms are still not a comprehensive explanation and are necessary to be explored.

### NK-related therapies

With the advent of cancer immunotherapy, targeting innate lymphoid cells has been reported. First, targeting inhibitory and activated NK cell receptors have been developed. Anti-KIR2D antibody (Ab) (Lirilumab; IPH2102) or combined with ICBs has been used to treat patients with hematological malignancies (Benson et al., 2012; Benson et al., 2015; Yalniz et al., 2018). An anti-NKG2A mAb (omalizumab; IPH2201) has been applied in chronic lymphocytic leukemia (André et al., 2018; Kamiya et al., 2019) and could unleash the cytotoxicity of NK by combining with anti-PD-L1 mAb (André et al., 2018). Besides, anti-NKG2A mAb is being evaluated in a clinical trial by combining an anti-EGFR Ab (cetuximab) in advanced solid cancers (NCT02643550). However, a study has shown that NKG2A blockade could promote CD8+T cell functions, but were ineffective for NK cells in mouse tumor model with HPV16 induction (van Montfoort et al., 2018). Additionally, CAR-NK cells have been engineered to have a chimeric receptor (NKG2D), which improves their cytotoxic capacity against tumor cells (Chang et al., 2013; Parihar et al., 2019). Second, a novel approach, using pluripotent stem cells (iPSC) to elicit NK cells, has been designed. Treatment with iPSC-derived NK cells or combined with anti-PD-1 Ab made cancer cell growth arrest (Li et al., 2018; Cichocki et al., 2020). Third, CAR-NK cell-based therapeutic regimens are considered as a promising therapeutic method, and increasing evidence has been shown in the preclinical models. The therapeutic strategy using CAR-NK cells has been proven to improve the anti-tumor efficacy in preclinical models of CRC and acute myeloid leukemia (AML) (Hayes, 2021). HER-2-specific CAR-NK cells were injected into ovarian cancer mice also ameliorated NK cytotoxicity (Han et al., 2015). CXCR1-modified NK cells enhanced anti-tumor activity in ovarian cancer mice with peritoneal xenografts (Ng et al., 2020). CAR-NK cells with targeting EGFR increased anti-tumor efficacy in a mouse model of glioblastoma (Han et al., 2015). CAR-NK cells with targeting CD19 can the cytotoxic activity of NK cells in acute lymphoblastic leukemia (ALL) (Quintarelli et al., 2020). Noteworthily, Cytomegalovirus (CMV), the most potent stimulator of NK cells, has been adopted to treat pediatric ALL and it could prolong the survival (Sivori et al., 2021). DAP10, when added to the CAR-NK cells, has been reported to enhance NK cell cytotoxicity potently through facilitating and maintaining the expression of NKG2D (Morvan and Lanier, 2016). Additionally, it is intriguing that cytokines also are considered to add to the frame of CAR-NK cells. For example, IL-15 incorporated into the CAR construct enhanced NK cell cytotoxicity and eliminated tumor cells (Daher and Rezvani, 2021). Although CAR-NK cells have been designed to combat tumor cells, there are few relevant studies. In recent years, in order to find out beneficial approaches, researchers have registered relevant clinical trials (NCT03415100, NCT03940820, NCT03692637, NCT02839954, NCT03383978, and NCT03941457). Of note, two clinical trials have been withdrawn and suspended, respectively (NCT03579927, NCT01974479).

Moreover, because cytokines are important for ILCs, cytokine-based therapy could affect the functions of ILCs. Pre-activated NK cells *ex vivo* by several cytokines, primarily including IL-12, IL-15, and IL-18, could be endowed with memory-like features, termed cytokine-induced memory-like NK cells (CIML-NK), and then last to exert an anti-tumor function (Romee et al., 2016). At present, this strategy has been investigated for hematological malignancies (NCT01898793, NCT03068819, and NCT02782546). TGF-β is a potently immunosuppressive factor. A study has been conducted that deleting TGFβR2 from NK cells using CRISPR-Cas9 technology could suppress the function of TGF-β and maintain their cytotoxicity in AML. Therefore, NK cells have been engineered to express a non-functional TGFβR2-like receptor in order to inhibit the function of TGF-β (Daher and Rezvani, 2021). High doses of IL-2 have been applied to the clinical practice to treat a small part of patients with advanced tumors (Marabondo and Kaufman, 2017), but IL-2 could increase the number of Tregs (Ghiringhelli et al., 2005; Adotevi et al., 2018). Furthermore, researchers found utilizing IL-15 did not result in Tregs expansion in patients with neuroblastoma (Nguyen et al., 2019). Consequently, IL-15 which increases the number and function of NK cells, is considered as a therapeutic strategy. Therapy with IL‐15 superagonist, ALT-803, has been reported to boost anti-tumor activity of NK and T cells and prolong patient survival (Hosseini et al., 2020; Sivori et al., 2021). It is surprising that ALT-803 can attach to other molecular structures in order to generate a pleiotropic compound and obtain benefits (Sivori et al., 2021). Recently, treatment with IL-15 has been investigated and the results of several clinical trials have been published (NCT01572493, NCT03759184, NCT03905135, NCT04185220, and NCT02689453). Treatment with the combination of human IL-15 (rhIL-15) and monoclonal antibody, including alemtuzumab, obinutuzumab, avelumab, or mogamulizumab, has been reported to boost the cytotoxicity of NK cells and enhance the efficacy of these monoclonal antibodies in small population patients with advanced chronic lymphocytic leukemia (Dubois et al., 2021). However, these studies have found that systemic IL-15 (N-803) impacted the presence of infused NK cells in AML, although it improved the function of CD8+ T cells (Berrien-Elliott et al., 2022; Pende and Meazza, 2022). Therefore, N-803 is still being investigated in the clinical trials (NCT03050216 and NCT01898793). Another cytokine, IL-12, is of a similar anti-tumor function to IL-15. The injection of membrane-bound interleukin 21 (mbIL-21) after haploidentical HSCT of patients with leukemia reduced the risk of relapse (Ciurea et al., 2017). Additionally, the combination therapy of IL-15 and IL-21 was used in rhabdomyosarcoma to enhance anti-tumor response (Wagner et al., 2017b).

Lastly, novel polyfunctional antibodies, termed natural killer cell engagers (NKCEs), have been generated. NKCEs have been proposed to generate a more effective benefit against tumor cells (Davis et al., 2015). The CD16 x CD33 NK cell engager was the first bispecific killer engager (BiKE) which are used to treat patients with AML (Wiernik et al., 2013). Furthermore, new tri-specific killer cell engagers (TriKE) have been designed. Anti-CD16 x IL-15 x anti-CD33 TriKE played an anti-tumor role through eliciting NK cell functions in mouse models of tumors (Vallera et al., 2016; Vallera et al., 2020), and its efficacy was reported in a terminated clinical trial (NCT03214666). Anti-CD16 x anti- CD19 x IL-15 TriKE promoted NK cells to perform tumoricidal functions in chronic lymphoid leukemia (Felices et al., 2019). Similarly, another tri-specific NK cell (1615133TriKE) also could eliminate tumor cells by the mechanism of ADCC (JU et al., 2017). Besides, human EGFR3 x NKp30 NK cell engagers have been developed, which is modified based on the affinity of B7-H6. They induced NK cells to secret cytokines and eliminate tumor cells (Demaria et al., 2021). NKp46-NKCEs fused with a tumor antigen and an Fc fragment could kill tumor cells by the mechanism of ADCC (Demaria et al., 2021). Intriguingly, adaptive NK cells with potent ADCC capacity were able to not only ablate the immunosuppressive response of MDSCs and Tregs, but also amplify the efficacy of BiKE and TriKE (Sivori et al., 2021). The various NKCE strategies are promising therapeutic tactics and are necessary to be further explored.

### The hILC-related therapies

At present, harnessing helper ILCs is relatively rare and mainly targets cytokines that influence the cytotoxicity of these cells. Treatment with IL-33 alone or the combination of IL-33 with PD-1 blockade boosted the cytotoxicity of hILC2s and anti-tumor activity in a mouse model of melanoma (Maggi et al., 2020; Jacquelot et al., 2021b). However, IL-33 may stimulate hILC2s to produce the immunosuppressive ectoenzyme CD73, thereby promoting tumor growth (Maggi et al., 2020). Targeting the hILC2–MDSC axis should be promising in APL. IL-13 is a key molecule in this axis. Targeting the IL-13 receptor on tumor cells has shown a good efficacy in glioma mouse model. Anti-IL-13R mAb was also used to treat patients with glioblastoma (Maggi et al., 2020). In a completed clinical trial, treatment with IL-4 PE38KDEL cytotoxin in patients with relapsed gliomas has shown to have a good prognosis (NCT00014677). The high levels of IL-4R also promote tumor growth, so targeting IL-4R has been designed. It is well known that using anti-IL-4R antibodies had a significant impact on a variety of tumors (Yang et al., 2012a; Seto et al., 2014). In patients with AML treated by allogeneic HSCT, IL‐22 secreted by ILC3s might forestall graft versus host disease (GVHD), and thus IL‐22 could be a feasible treatment option (Munneke et al., 2014). The function and plasticity of helper ILCs are important for tumor therapy. Thus, increasing research into helper ILCs should be conducted in the future.

### Other tumor-infiltrating immune cells

#### Myeloid-derived suppressor cells

Myeloid-derived suppressor cells have been reported as inhibitors of anti-tumor immunity by antigen-specific and non-specific patterns (Serafini et al., 2006). Some researchers have shown that MDSCs are negatively associated with the prognosis of tumor patients (Serafini et al., 2006; Tian et al., 2019). MDSCs can be devided into two subtypes, monocytic MDSCs (M-MDSCs) and polymorphonuclear MDSCs (PMN-MDSCs). M-MDSCs could promote the maturation of DCs, differentiate into M2-TAM, produce nitric oxide (NO), Arg-1 which could deplete arginine, and secrete inhibitory cytokines including IL-10 and TGF-β (Wilczyński and Nowak, 2012; Tie et al., 2022a). PMN-MDSCs mainly induce antigen-specific T-cell tolerance and hinder T-cell migration by producing reactive oxygen species (ROS) (Gabrilovich, 2017; Li et al., 2020b). PMN-MDSCs also secrete some cytokines to facilitate angiogenesis in TME (12). Moreover, tumor cells could secrete various molecules to attract MDSCs into TME like GM-CSF and IL-6, in turn, MDSCs induce the mutations of tumor cells and express some proteins like SA100A8/9 to avoid immune surveillance (Sinha et al., 1950a; Bresnick et al., 2015; Li et al., 2020b). IL-6 also promotes MDSC accumulation and inhibits anti-tumor immunity by activating the JAK/STAT3 signaling pathway, which results in increased production of ROS, NO, and PD-L1 (Ostrand-Rosenberg and Fenselau, 1950; Weber et al., 2021). Other molecules such as CCR2 or CCR5 are important for the migration of MDSCs. MDSCs also induce the production of Tregs and Th17 cells (Sinha et al., 1950b; Messmer et al., 2015).

#### MDSC-based therapies

According to MDSC functions, targeting MDSCs has been developed. First, depletion of MDSCs has been carried out in mouse models. It is reported that tyrosine kinase inhibitors (TKIs) could deplete MDSCs. For instance, Sunitinib was used to eliminate MDSCs by interfering with VEGF and STAT3 signaling pathways in renal cell carcinoma (Peng et al., 2021b). Using ibrutinib could restrain the production MDSCs in melanoma (Stiff et al., 2016). Another novel therapeutic strategy has been proposed. Targeting S100A family proteins, “peptibodies” adjoined to antibody Fc fragments could selectively eliminate MDSCs (Qin et al., 2014). The TNF-related apoptosis-induced ligand (TRAIL) receptors are also considered as a target for the depletion of MDSCs (Condamine et al., 2014; Dominguez et al., 2017; Hartwig et al., 2017). Targeting TRAIL-R2, DS-8273a, is ongoing in advanced solid tumors and lymphoma (NCT02076451). Second, it is a practical strategy that MDSCs are blocked to migrate to the tumor sites. Targeting chemokines or its receptors which help MDSC migration could inhibit MDSC recruitment and trafficking. Targeting CCR5 secreted by MDSCs has been reported to reduce the number of tumor-infiltrating MDSCs, and improve the survival in melanoma and breast cancer (Velasco-Velázquez et al., 2012; Zhang et al., 2013; Blattner et al., 2018). Targeting CXCL13 or its receptor CXCR5 could decrease the accumulation of MDSCs in TME in preclinical models (Ding et al., 2015; Garg et al., 2017). CXCR2 antagonists have been reported to make MDSCs range from overt infiltration to subtle infiltration and T-cell infiltration increase in the tumor sites. Targeting CXCR2 also augments the therapeutic efficacy of PD-1 blockade (Highfill et al., 2014; Zhang et al., 2020). Additionally, CXCR2 antagonists (Reparixin and AZD5069) are currently in the clinical trial phase for locally advanced or metastatic breast cancer metastatic (NCT05212701) and castration-resistant prostate cancer (NCT03177187), respectively. Targeting CCL2–CCR2 axis has shown a good outcome in mouse models (Li et al., 2017; Xu et al., 2021b), but anti-CCL2 mAbs or CCR2 antagonists are mainly used to treat immune diseases in the clinical trials. Only a CCR2/CCR5 Dual Antagonist (BMS-813160) is being used to try to treat patients with locally advanced PDAC, which is in the clinical trial phase (NCT03767582). Targeting colony-stimulating factor 1 receptor (CSF1R) or its ligand CSF-1 could prevent myeloid cell differentiation into MDSC and impede tumor progression (Cannarile et al., 2017; Yeung et al., 2021). Other studies have demonstrated that CSF-1R blockades combined with CXCR2 antagonists, ICB or anti-VEGFR mAbs have better efficacy in tumor patients (Tie et al., 2022b). Third, the downregulation of immunosuppressive functions of MDSCs is a promising approach. The inhibition of COX-2/ PGE2 signaling could suppress MDSC functions, which leads to impairing the production of Arg-1 and ROS, improves CD8+ T cytotoxicity, and delays tumor growth (Eruslanov et al., 2010; Obermajer et al., 2012; Zelenay et al., 2015). In preclinical models of glioma, targeting COX2 combined with acetylsalicylic acid downregulated the levels of PGE2 and inhibited glioma progression (Fujita et al., 2011). Phosphodiesterase-5 (PDE-5) inhibitors could also impair the level of Arg-1 produced by MDSCs. Using PDE-5 inhibitors has been shown to boost the anti-tumor immune activity of T cells and NK cells, reduce the accumulation of MDSCs and Tregs, and promote cancer cell growth arrest in patients with HNSCC and metastatic melanoma (Weed et al., 2015; Hassel et al., 2017). The inhibition of the STAT3 pathway is another therapeutic strategy to impair the function of MDSCs. The STAT3 inhibitor, AZD9150, combined with ICB, has been utilized to treat patients with diffuse large B‐cell lymphoma in a clinical trial (NCT01563302). In localized and metastatic castration-resistant prostate cancer patients, treatment with TLR9-targeted STAT3 siRNA delivery to abrogate the immunosuppressive function of MDSCs diminished the enzymatic activity of Arg-1, inhibited STAT3 target gene and T cell function (Hossain et al., 2015). IL-6 inhibitors also impacted the STAT3 signaling by reducing STAT3 phosphorylation and the expression of STAT3 downstream anti-apoptotic genes in ovarian cancer (Guo et al., 2010a; Guo et al., 2010b). Lastly, another credible strategy is to reduce the production of MDSC populations. All-trans-retinoic acid (ATRA) binding to the retinoid receptor could induce the immature myeloid cell (IMC) population to differentiate into macrophages and dendritic cells, neutralize high ROS production and increase glutathione synthase (Liu et al., 2021b; Bi et al., 2022). ATRA administration or combined with other immunotherapies increased the number of T cells, enhanced dendritic cell functions, and downregulated the ROS generation in MDSCs, resulting in improving anti-tumor immunity (Li et al., 2014; Bauer et al., 2018; Tobin et al., 2018). The combination of ATRA and ipilimumab are more effective than using ipilimumab monotherapy alone in metastatic melanoma and cervical cancer patients (Tobin et al., 2018; Liang et al., 2022). ATRA administration is an extremely promising therapeutic option for restraining the immunosuppressive functions of MDSCs, thus, its application needs to be further explored in other tumors. Additionally, some studies have reported that histone deacetylase inhibitors (HDACs) also control the differentiation of MDSCs and inhibit MDSC functions in tumor mouse models (Orillion et al., 2017; Briere et al., 2018; Christmas et al., 2018). The low-dose HDACi trichostatin-A could impair the suppressive activity of MDSCs and prevent MDSCs from trafficking, but the off-target effects that is the upregulation of PD-L1 should be tackled in further research (Li et al., 2021; Adeshakin et al., 2022). Other approaches to impact the differentiation and function of MDSCs, such as promoting the expression of interferon regulatory factor (IRF)-8, and inhibiting casein kinase 2 (CK2) signaling, are promising strategies (Valanparambil et al., 2017; Hashimoto et al., 2018; De Cicco et al., 2020; Xia et al., 2020).

In summary, targeting MDSCs have been developed and acquired good outcomes in preclinical models. However, due to the MDSC heterogeneity in various tumor types, the drugs targeting MDSCs could not be applied broadly. Thus, further studies are indispensable in the different tumor types. Secondly, the plasticity of MDSCs has been mentioned previously. Thus, the factors which reshape the differentiation of MDSCs are essential for future treatment strategies. In addition, even if the depletion of MDSC and the inhibition of MDSC trafficking are favorable options, the complicated mechanisms to reduce the number of MDSCs have not been revealed, so complementary researches are cardinal to develop new options and improve the prognosis.

#### Tumor-associated macrophages

Macrophages have been traditionally divided into two types: inflammatory M1-macrophages (anti-tumoral phenotypes) and immunosuppressive M2-macrophages (pro-tumoral phenotypes) (Cassetta and Pollard, 2020; Christofides et al., 2022). Macrophages are recruited into the tumor sites and play different functions, termed tumor-associated macrophages. CSF1 is a key factor for the recruitment of macrophages and polarizes macrophages to express the M2 phenotype (De et al., 2016; Christofides et al., 2022). It is reported that the inhibition of CSF-1 could decrease the accumulation of TAM and transform the M2 phenotype into the M1 phenotype (Ramesh et al., 2019). CCL2 also attracts macrophages into the tumor sites and mediates macrophage polarization (Korbecki et al., 2020; Yang et al., 2020). TAMs have a dual function, pro-tumoral and anti-tumoral functions. TAMs promote tumor progression by following pathways. First, TAMs release various molecules to assist tumor cell proliferation and metastasis. Growth factors expressed by TAMs such as epidermal growth factor (EGF) aid tumor cell proliferation. NF-kB-mediated factors like IL-6 and CCL2 prevent tumor cell apoptosis (Xiang et al., 2021). TAMs also induce and activate the Wnt/β-catenin signaling, resulting in the proliferation of tumor progenitor cells in liver cancer (He and Tang, 2020). TAMs secrete CCL5 to activate the STAT3β-catenin pathway and favor the metastasis of tumor cells (Huang et al., 2020). The proangiogenic growth factors released by TAMs like VEGF, platelet-derived growth factor (PDGF) and fibroblast growth factor (FGF) also facilitate tumor cell migration (Kumari and Choi, 2022). Second, TAMs play an immunosuppressive role by expressing various small molecules. TMAs facilitate the ICs expression on tumor cells or produce IL-10, TGF-β, Arg-1, and IDO to impede T cell function (Arlauckas et al., 2018; Xiang et al., 2021; Kumari and Choi, 2022). Finally, TAMs also upregulate the level of inhibitory receptors to inhibit T cell and NKcell activity and recruit Tregs into TME (Yang et al., 2012b; Wu et al., 2019; Tie et al., 2022a). Conversely, TAMs could inhibit tumor progression by increasing their phagocytic capacity and enhancing the function of antigen presentation. TAMs also produce cytokines to activate Th1 and CD8+T cells and improve anti-tumor immunity (Moeini and Niedźwiedzka-Rystwej, 2021). Of note, the polarization of macrophages could be reshaped in the complex TME. For instance, activated mTOR signaling pathways facilitated the polarization of M2 macrophages (Mazzone et al., 2018; Chen et al., 2022).

#### The TAMs-based immunotherapies

Targeting TAMs mainly inhibits its pro-tumoral functions, and several studies have been conducted. First, depleting TAMs and arresting the recruitment of TAMs have been reported. Targeting the CSF-1/CSF-1R axis has been tested in preclinical models and is a significant therapeutic option to decrease the production and aggregation of TAMs. Inhibiting the CCL2/CCR2 axis is another reasonable treatment strategy for preventing TAMs from migrating into tumor sites. The efficacy of CSF-1 and CCL2 inhibitors has been comprehensively reviewed, therefore, we will not be covered here (reviewed in refs (Rasmussen and Etzerodt, 2021)). Second, inhibition of the immunosuppressive function of TAMs could boost anti-tumor immunity by polarizing M2 TAMs into anti-tumor phenotypes. The TLR7/8 influences the TAM polarization to skew toward M1 TAM, thus, its agonist, R848-Ad, has been used to treat tumors in the mouse models and then improves the anti-tumor activity (Rodell et al., 2018; Rodell et al., 2019). Upon a TLR agonist, targeted delivery of a long peptide antigen to TAMs *via* using a nano-sized hydrogel (nanogel) activated TAMs to promote tumor apoptosis and activate anti-tumor immune responses, including antigen-presenting activity and altering tumor immune responses from resistance to responsiveness (Muraoka et al., 2019; Tie et al., 2022a; Zhang et al., 2022b). Activated TLR3 ligands also shift the M2 phenotype to the M1 phenotype by upregulating the expression of MHC-II molecule (Vidyarthi et al., 2018). The PI3K/AKT pathway is responsible for the recruitment of M2-TAM, thus, PI3Kα inhibitors could impede tumor cell growth and invasion (Khan et al., 2013). PI3Kγ upregulates the immunosuppressive properties and downregulates the anti-tumor properties of TAMs (Vergadi et al., 1950; Zhang et al., 2017b; Kaneda et al., 2017), thus, targeting PI3Kγ would be necessary. Activation of the CD40 receptor on TAMs could convert TAMs into M1 macrophages (Wiehagen et al., 2017; Hoves et al., 2018). Using the combination of CD40 agonists and anti-CSF1R antibodies enhanced the cytotoxicity of T cells (Xiang et al., 2021). The inhibition of the NF-κB pathway by the siRNA pathway could transform TAM into M1 macrophages (Ortega et al., 2016). Other molecules also impact the polarization of TAMs, such as HDAC, and the microRNA processing enzyme DICER, because blocking HDAC or DICER could produce M1 phenotypes (Tie et al., 2022a; Tajaldini et al., 2022). Third, restoring phagocytic capacity is crucial for TAMs. The activated SIRPα–CD47 axis could limit the TAM phagocytic capacity for cancer cells. Therefore, targeting SIRPα has been proposed and tested in pancreatic cancer and breast cancer, where it strengthens the phagocytosis ability of macrophages and promotes tumor cell death (Jaiswal et al., 2009; Theocharides et al., 2012; Hu et al., 2020; Jia et al., 2021). Anti-CD47 antibodies have also been reported to strengthen the anti-tumor activity of macrophages (Brierley et al., 2019; Sikic et al., 2019; Jia et al., 2021), and some anti-CD47 mAbs in different tumors are in the clinical trials (NCT02953509, NCT04751383). Inhibition of leukocyte immunoglobulin-like receptor subfamily B (LILRB)- MHCI pathway axis could recover the phagocytic capacity of TAMs (Barkal et al., 2018). Finally, CAR expressed by TAMs has been reported to improve phagocytosis, transform TAMs to the M1 phenotype, and enhance anti-tumor immunity (Christofides et al., 2022). CAR for phagocytosis (CAR-P) could improve the phagocytic capacity of TAMs and hinder tumor cell growth in solid tumors (Morrissey et al., 2018; Kumari and Choi, 2022). CAR-macrophages (CAR-M) have also been developed, which inhibits tumor progression in a mouse model (Klichinsky et al., 2020; Villanueva, 2020; Sloas et al., 2021). Furthermore, a recent study proposed another tactic, anti-CCR7 CAR-M cells, which induces macrophages toward CCR7-positive cells and then deletes CCR7-positive cells by a series of activities (Kumari and Choi, 2022). At present, a novel method, targeting TAMs with nanomaterials, has been reported. The use of nanomaterials to inhibit tumor growth not only promotes anti-tumor immunity, but it also reduces the off-target effects and adverse events. The details of nanoimmunotherapies for TAMs have been concluded in this review (Kumari and Choi, 2022). Therefore, targeting nanoimmunotherapies is a promising option. Thus, more research is needed in the future.

In brief, although these therapeutic strategies have been successfully applied in mouse models and even in the clinical trials, targeting TAMs is still limited. At present, targeting all macrophage populations is less effective than targeting pro-tumor TAMs, so researchers want to further focus on targeting a specific macrophage population to reach maximal efficacy. Additionally, though the blockade of recruitment of TAMs is a potential option, it is a better strategy that converts TAMs from M2 to M1. Thus, further exploration needs to dissect the mechanisms of TAM polarization from top to bottom and develop novel treatment agents. Moreover, some inhibitory molecules have not been thoroughly discussed, including CD163, CD206, and TREM2. Thus, extensive investigations about targeting these molecules needs to be performed. Finally, the combination therapy of based-TAM and other ways like radiotherapy are also worthy of research.

## Conclusion and perspectives

**FIGURE 2:**
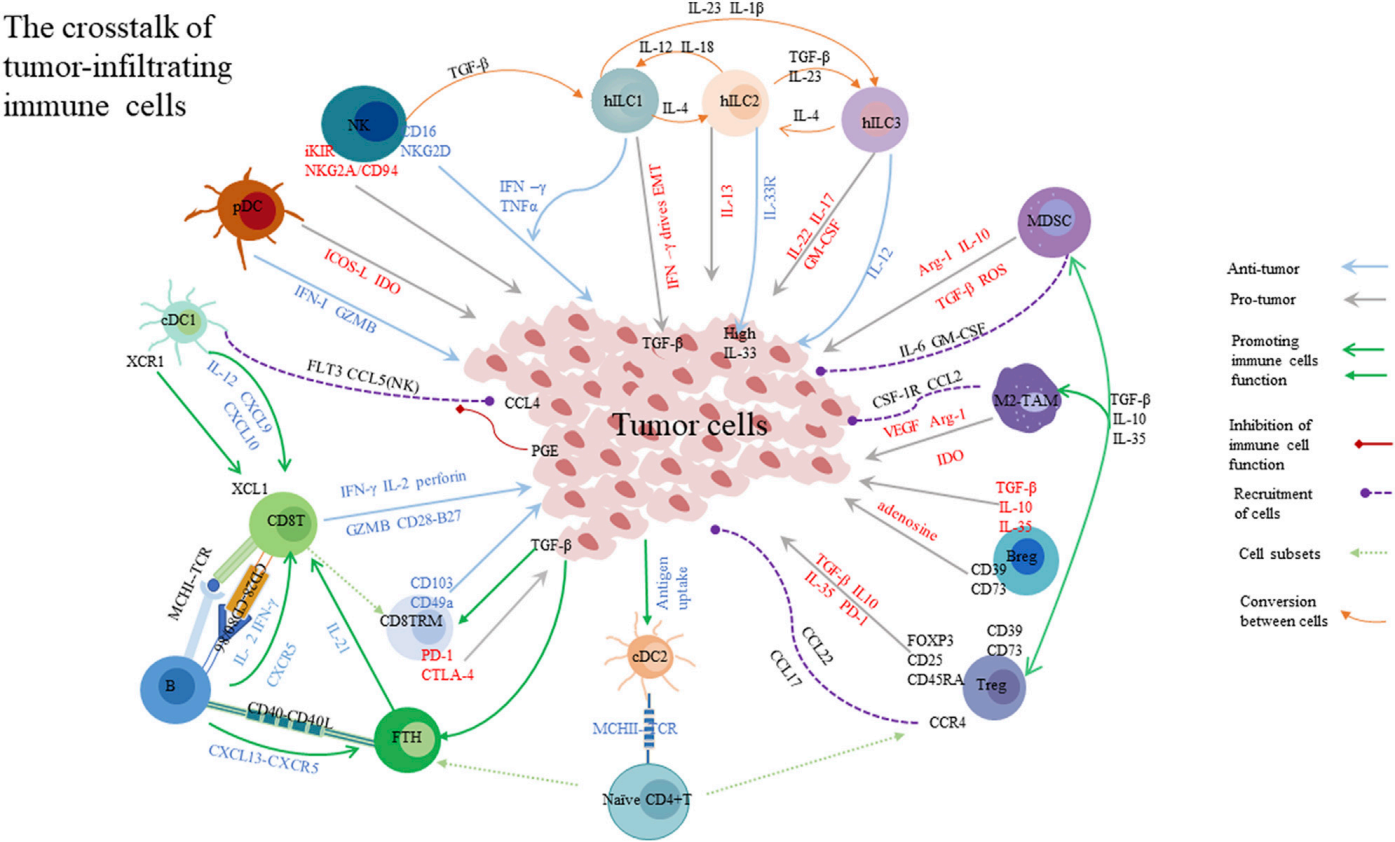
Tumor-infiltrating immune cells are important. Different cells play different roles. CD8+ T cell, CD8 TRM, and NK could kill tumor cells. Bregs, Tregs, MDSCs, and M2-TAM promote tumor cell growth. Tumor cells also secrete various molecules to disturb immune cell function. These molecules can convert cell phenotype and change their function, like NK cells. Specially, TGF-β derived from tumor cells could promote the function of CD8 TRM and Tfh cells. The crosstalk of these immune cells are is important for their function. Inhibitory cells secrete various immunosuppressive molecules to impair the cytotoxicity of effector cells. CD8 TRM: CD8 tissue resident memory; DC: dendritic cell; cDCs: conventional dendritic cells; pDCs: plasmacytoid DCs; Tfh: T follicular cell; NK: natural killer; hILC: helper innate lymphoid cells; Bregs: regulatory B cells; Tregs: regulatory T cells; MDSC: myeloid-derived suppressor cell; M2-TAM: M2 macrophages; EMT: epithelial mesenchymal transition.

With the advancement of immunotherapies, it is well revealed that tumor-infiltrating immune cells play increasingly important roles and interact with the efficacy of targeting these immune cells. Therefore, the dissection of these cell properties in TME is indispensable for improving the clinical response of immunotherapies. In this review, we have dissected the characteristics of mainly tumor-infiltrating immune cells, including their phenotypes, their recruitment, their activation, and immune-based therapies (Figure 2). The interactions with these cells in TME are complicated due to the presence of anti-tumor and pro-tumor activities, which is associated with the clinical outcome of cancer patients. In addition, these cells also form a special structure which impacts the clinical outcome of tumor patients, but it remains in its infancy for TLS properties. Correspondingly, according to the characteristics of these immune cells, various immunotherapy approaches have been developed and are successful in preclinical tumor models and human tumors, including targeting cytokines and chemokines, targeting various phenotypes, and CAR-T. Recently, nanoimmunotherapies have been generated and acquired a better efficacy, which provides a novel approach to target tumor cells and is worthy of emulation. However, due to the conspicuously complex TME, the clinical response of some strategies is limited, even ineffective and resistant. Consequently, extensive complementary researches on tumor-infiltrating immune cells are necessary to overcome these shortcomings and further develop curative tactics for a conducive prognosis.

## Data Availability

The original contributions presented in the study are included in the article/Supplementary Material, further inquiries can be directed to the corresponding authors.
